# Progress in Anti-Mammarenavirus Drug Development

**DOI:** 10.3390/v13071187

**Published:** 2021-06-22

**Authors:** Yu-Jin Kim, Victor Venturini, Juan C. de la Torre

**Affiliations:** 1 Department of Immunology and Microbiology, The Scripps Research Institute, La Jolla, CA 92037, USA; yujin@scripps.edu (Y.-J.K.); v.venturini@outlook.es (V.V.); 2 Department of Biotechnology, Faculty of Experimental Sciences, Francisco de Vitoria University (UFV), Carretera Pozuelo-Majadahonda, Km 1,800, Pozuelo de Alarcón, 28223 Madrid, Spain

**Keywords:** mammarenavirus, antiviral drug, drug repurposing, high-throughput screening

## Abstract

Mammarenaviruses are prevalent pathogens distributed worldwide, and several strains cause severe cases of human infections with high morbidity and significant mortality. Currently, there is no FDA-approved antiviral drugs and vaccines against mammarenavirus and the potential treatment option is limited to an off-label use of ribavirin that shows only partial protective effect and associates with side effects. For the past few decades, extensive research has reported potential anti-mammarenaviral drugs and their mechanisms of action in host as well as vaccine candidates. This review describes current knowledge about mammarenavirus virology, progress of antiviral drug development, and technical strategies of drug screening.

## 1. Introduction

Members of the family *Arenaviridae* are classified into four genera based on phylogenetic analysis of their RNA-directed RNA polymerase (L protein) and nucleoprotein (NP) sequences: *Antennavirus*, *Hartmanivirus*, *Mammareanvirus*, and *Reptarenavirus* [1]. Antennaviruses (2 species) were discovered in actinopterygian fish by next-generation sequencing, and no biological isolate has been reported yet. Hartmaniviruses (4 species) and reptarenaviruses (5 species) infect captive snakes, and some of them have been associated with boid inclusion body disease (BIBD). Mammarenaviruses (39 species) infect mainly rodents, and the infection is generally asymptomatic. Current knowledge about the biology of snake and fish arenaviruses is very limited, and their zoonotic potential unknown. In contrast, some mammarenaviruses have been found to infect and cause disease in humans.

Mammarenaviruses are enveloped viruses with a bi-segmented single-stranded negative-sense RNA genome [1]. Mammarenaviruses cause chronic infections of their rodent natural reservoirs across the world, but some of them have zoonotic potential. Human infections occur through mucosal exposure to aerosols or by direct contact of abraded skin with infectious materials [2]. Based on their antigenic properties, mammarenaviruses have been classified into two distinct groups, Old world (OW) mammarenaviruses, aka Lassa-lymphocytic choriomeningitis virus serocomplex,” including viruses present in Africa and the worldwide distributed lymphocytic choriomeningitis virus (LCMV), and the New World (NW) mammarenaviruses, aka “Tacaribe serocomplex”, including viruses indigenous to the Americas [3]. Both OW and NW mammarenaviruses include several species members that can cause severe hemorrhagic fever (HF) diseases in humans that are associated with high morbidity and significant mortality; these viruses include Lassa (LASV), Junin (JUNV), Machupo (MACV), Guanarito (GTOV), Sabia (SABV), Chapare (CHPV), and Lujo (LUJV) [4]. Concerns posed by human pathogenic mammarenaviruses are exacerbated by the overall lack of FDA-licensed vaccines and current anti-mammareanavirus therapy being limited to off-label use of ribavirin that is only partially effective, has a narrow therapeutic window, and can be associated with side effects [5]. The only mammarenavirus vaccine tested in humans is the live-attenuated Candid#1 strain of JUNV that has been shown to be safe and provide effective protection against Argentine HF (AHF) disease caused by JUNV [6,7]. Accordingly, Candid#1 is approved in Argentina for use in populations at high risk of JUNV infection [8].

This article presents a concise review of our current understanding of the mammarenavirus life cycle at the molecular and cellular level and of progress on antiviral drugs targeting specific steps of the mammarenavirus life cycle and their implications for potential therapeutic strategies against human pathogenic mammarenaviruses.

## 2. Mammarenavirus Molecular and Cell Biology

### 2.1. Virus Particle, Viral Proteins and Genomic Organization

**Figure 1:**
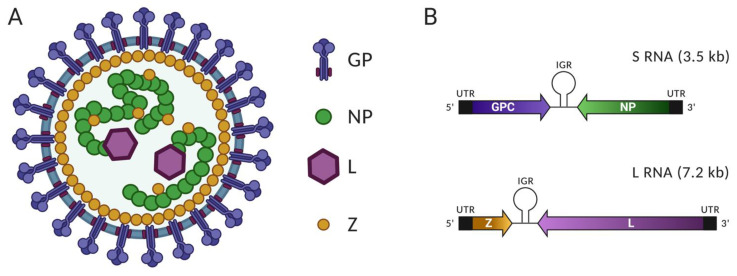
Mammarenavirus virion and genome organization. (**A**) Schematic diagram of mammarenavirus virion. The virion is enveloped and contains four types of viral proteins; glycoprotein (GP), nucleoprotein (NP), RdRp (L), the RING finger protein Z. (**B**) Genome organization of mammarenaviruses. The bi-segmented negative sense RNA genome consists of the large (L) and small (S) segments. This ambisense genome organization encodes two independent viral proteins in each segment; GPC and NP in the S segment, Z and L in the L segment.

Mammarenavirus virions are spherical lipid-enveloped particles with a wide diameter range (400 to 2000 Å) that enclose the bi-segmented negative-stranded (NS) RNA viral genome [9]. Each genome segment, L and S, uses an ambisense coding strategy to direct the synthesis of two polypeptides [10]. The small (S, ca 3.5 kb) genome segment encodes the viral nucleoprotein (NP) and the glycoprotein precursor GPC that, upon co-and post-translational processing, generates the GP complex that forms the spikes present at the virion surface and that mediate virus cell entry. The large (L, ca 7.5 kb) genome segment encodes the viral RNA-dependent RNA polymerase (RdRp; L protein) and the small RING finger protein Z that is a bona fide matrix protein (Figure 1A). NP is the most abundant viral protein in infected cells and virions. NP encapsidates the virus genome and antigenome RNA species to form a nucleocapsid complex that, in association with the viral L polymerase, forms the viral ribonucleoprotein complex (vRNP) that directs the biosynthetic processes of replication and transcription of the viral genome [11]. NP has also been shown to counteract the induction of type I interferon (IFN-I) response that plays a critical role in the cell innate immune response to infection [10,12,13,14]. This anti-IFN-I activity of NP has been linked to a functional DEDDh 3′–5′ exoribonuclease domain present within the NP C-terminal region [15,16]. GPC is co-translationally cleaved by cellular signal peptidases to generate a 58 amino acid-long stable signal peptide (SSP) and the GP1/2 precursor that is post-translationally processed by the cellular protease subtilisin kexin isozyme-1 (SKI-1)/site 1 protease (S1P) to generate the mature virion surface glycoproteins GP1 and GP2 [17]. The SSP, together with GP1 and GP2, form the spikes (GP complex) located at the surface of mature virions and that mediate virus cell entry. The GP1 subunit is located at the top of the spike away from the membrane and mediates virion interaction with host cell-surface receptors [18,19]. The GP2 subunit is involved in virion-cell membrane fusion by means of a conformational change triggered by the acidic environment of the endosome [20,21]. The SSP has been implicated in the trafficking and processing of the viral envelope glycoproteins and in the GP2-mediated pH-dependent fusion process [3]. N-glycosylation modifications of GPC are required for SKI-1/S1P cleavage [22], whereas SSP must be myristoylated for the GP2-mediated fusion activity [23]. The L protein mediates replication and transcription of the virus RNA genome by the vRNP. The L protein also has an endonuclease activity that mediates the cap-snatching process required for transcription initiation [11,24]. The RING finger protein Z serves as a bona fide matrix protein [25]. Z has been shown to interact with various host cell proteins, including the eukaryote translation initiation factor 4E (eIF4E) and the promyelocytic leukemia protein (PML), interactions that might contribute to facilitate virus multiplication [26,27,28,29,30]. As with other matrix proteins of NS RNA viruses, Z also negatively regulates the activity of the virus polymerase [31,32], mediates interactions between the vRNP and GP2 required for assembly of matured viral particles [33,34], and is the main driving force of viral budding from the plasma membrane, a process that includes Z’s interaction with several members of the endosomal sorting complex required for transport (ESCRT) [25,35]. Z has also been shown to interact with the cytosolic pathogen recognition receptor (PRR) RIG-I and counteracts the induction of the IFN-I response.

Both genome segments contain non-coding cis-regulatory elements required for replication and transcription of the virus genome, including the 5′ and 3′ untranslated regions (UTR) present at the ends of both RNA segments and the non-coding intergenic regions (IGR) separating the two open reading frames present in each genome segment [36]. Mammarenaviruses have highly conserved sequences at the 3′-end of the L and S RNA genome segments that contain the genome promoter recognized by the virus polymerase to initiate RNA synthesis [37]. Genomes and antigenome RNAs are highly complementary between their 5′- and 3′-termini, with both L and S genome segments, predicted to form panhandle structures, which is consistent with EM images showing the existence of circular RNP complexes within mammarenavirions. Mammarenavirus IGRs are predicted to fold into stable hairpin structures that constitute bona fide transcription termination signals. There are significant differences in sequence and predicted folded structure between the S and L IGR, but among isolates and strains of the same arenavirus, the S and L IGR sequences are highly conserved. The IGR has been also implicated in virus assembly or budding, or both, being required for the generation of infectious particles [38,39].

### 2.2. Mammarenavirus Life Cycle

#### 2.2.1. Cell Entry

**Figure 2:**
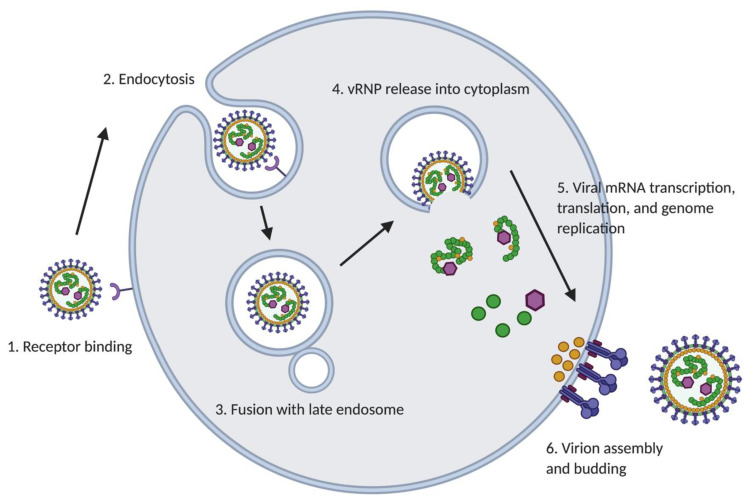
Mammarenavirus life cycle. Virus cell entry is initiated by the interaction between GP and different receptors present at the cell surface (1). Virus uptake into the cell is mediated by endocytosis (2). The acidic environment of the late endosome promotes fusion between viral and host cell membranes (3). Following the pH-dependent membrane fusion event, the vRNP is released into the cytoplasm, where it directs the biosynthetic processes of replication and transcription of the viral genome (4,5). Viral assembly takes place in the cell cytoplasm, and virions bud from the plasma membrane. Z plays critical roles in both these processes (6).

Mammarenavirus enter susceptible cells mainly via receptor-mediated endocytosis. The acidic environment of the late endosome facilitates a pH-dependent conformational change in the GP complex that induces a GP2-mediated fusion step between viral and cell membranes. Following fusion, the viral RNP is released into the cytoplasm, where it directs both replication and transcription of the viral genome (Figure 2). Different cell surface receptors are used by Old World (OW) and New World (NW) mammarenaviruses. OW mammarenaviruses, including lymphocytic choriomeningitis virus (LCMV) and Lassa virus (LASV), as well as clade C NW mammarenaviruses, use α-dystroglycan as the major high-affinity receptor to which GP1 attaches [40,41]. In contrast, pathogenic clade B NW mammarenaviruses including Guanarito (GTOV), Sabia (SABV), Junin (JUNV), Machupo (MACV) viruses use human transferrin receptor 1 (hTfR1) as the main receptor for entry [42]. A cell entry receptor for clade A NW mammarenaviruses has not been identified yet [43]. The OW hemorrhagic fever mammarenavirus Lujo virus (LUJV) LUJV was shown to use neuropilin (NRP)-2 as its main cell entry receptor [44]. Interestingly, NRP-2 is highly expressed on microvascular endothelial cells and alveolar macrophages, which may explain the extent of coagulopathy observed in LUJV-induced clinical disease and aerosol transmission of LUJV [45,46,47]. Once GP1 binds to the cellular receptor, the virion is endocytosed following different pathways by NW and OW mammarenaviruses. NW mammarenaviruses are internalized via clathrin-dependent endocytosis [48,49], whereas OW arenaviruses proceed through a clathrin-independent pathway that involves PI3K-mediated formation of multivesicular bodies (MVB) during late endocytosis and the endosomal sorting complex required for transport (ESCRT) proteins [50]. Notably, completion of the cell entry process for LASV and LUJV requires a late endosomal receptor switch mechanism. For LASV, the lysosome-associated membrane protein 1 (LAMP1) mediates a cholesterol-dependent pH-receptor switch mechanism [51], which stimulates GP1 dissociation from the GP complex and a conformational change in GP2 that drives the fusion of viral and cell membranes [20,52]. In the case of LUJV, this process is mediated by the tetraspanin CD63 [44].

#### 2.2.2. Genome Replication and Transcription

Following its release from the late endosome into the cytoplasm, the vRNP directs the biosynthetic processes of replication and transcription of the viral genome RNAs (Figure 2). L and NP are the minimal viral trans-acting factors required for these processes [53,54], whereas Z has been shown to exhibit a dose-dependent inhibitory effect on the vRNP activity, likely by interacting with the viral L polymerase and disabling its catalytic activity [31,53].

Mammarenaviruses generate three different RNA species during replication and transcription: genomic (gRNA) and antigenomic (agRNA) RNA species and sub-genomic viral mRNA species. Synthesis of viral mRNAs starts at the genome promoter and terminates within the distal region of the hairpin-structured IGRs [4]. Due to the ambisense coding strategy that characterizes mammarenavirus genome, NP and L mRNAs are transcribed from the genomic RNA, while GPC and Z mRNAs are transcribed from the corresponding antigenome RNA species. This ambisense coding strategy has been suggested to produce a hierarchal pattern of gene expression where NP and L proteins are produced at very early times of infection, and GPC and Z proteins at later times [4,55].

Mutation-function analysis using cell-based minireplicon assays for several mammarenaviruses indicated that the activity of the mammarenavirus genomic promoter requires both sequence specificity within the highly conserved 3′-terminal 19 nucleotides of arenavirus genomes and the integrity of the predicted panhandle structure formed via sequence complementarity between the 5′- and 3′-termini of viral genome RNAs [37,56]. IGRs serve as bona fide transcription termination signals, but the synthesis of translation-competent viral mRNAs does not strictly require the presence of the IGR [38,39,57].

Mammarenavirus transcription initiation uses a cap-snatching mechanism, where short 5′-capped primers derived from host-cell mRNAs are used by the virus polymerase complex to prime the synthesis of viral mRNAs [4,58]. The 5′-capped primers are generated via cleavage of cellular mRNAs by the endonuclease activity associated with the N-terminus of L [59]. The 5′-end of mammarenavirus genome and antigenome RNAs each contain a non-templated G residue that likely reflects a prime-and-realign mechanism for RNA replication mediated by L [60,61]. Mammarenavirus genome and antigenome, sequence terminal complementarity combined with the prime-and-realign mechanism for replication initiation generates double-stranded RNAs with overhanging 5′-ppp nucleotides. These structures can act as RIG-I decoys, thereby diminishing RIG-I-mediated interferon induction in mammarenavirus-infected cells [62]. NP mRNA of the NW mammarenavirus Tacaribe (TCRV) was detected at early times of infection and in the presence of inhibitors of protein synthesis, suggesting that, unlike the closely related bunyaviruses, but similarly to orthomyxoviruses, rhabdoviruses, and paramyxoviruses, primary transcription directed by the incoming mammarenavirus vRNP can proceed in the absence of translation [4,63].

#### 2.2.3. Assembly and Budding

As with many other enveloped NS RNA viruses, mammarenavirus assembly and budding require the participation of the matrix protein Z [64]. Z interacts with GP2 [33] and NP [65], which facilitates vRNP-GP complex interactions required for the assembly of mature viral particles (Figure 2). As with other matrix proteins, Z has the capability to self-assemble and direct budding of VLPs [35]. Z budding activity is driven by canonical viral late (L) domains within the C-terminus of Z, which mediate Z interaction with components of the cellular ESCRT complexes [66] known to play essentials roles in the budding process of different viruses [67,68].

## 3. Drugs Targeting Different Steps of Mammarenavirus Life Cycle

### 3.1. Cell Entry

Completion of mammarenavirus cell entry requires a GP2-mediated fusion event between viral and cellular membranes, a process triggered by the acidic environment of the late endosome. Therefore, targeting the GP2-mediated fusion event is an attractive strategy to inhibit mammarenavirus infection. Accordingly, a high-throughput screen (HTS) of a small molecule library identified a series of molecules (ST-193, ST-294, and ST-336) that target GP2 and inhibit viral entry [69,70]. ST-193 has been shown to confer significant protection against LASV infection in a guinea pig model [71], and the optimized chemical analog of ST-193 compound, LHF-535, was shown to be a potent broad-spectrum inhibitor of HF mammarenaviruses via targeting the GP2-mediated fusion step of virus cell entry [72]. Another GP2-mediated fusion inhibitor, F3406, was shown to inhibit LCMV multiplication [73]. AVP-p, a peptide derived from the Pichinde virus (PICV) GP2 subunit, was found to bind the prefusion state of the GP complex, which arrests the GP2-mediated fusion event [74]. Arbidol (umifenovir) was developed as an inhibitor of influenza virus infection, but it has been shown to exhibit broad-spectrum antiviral activity against different viruses by interfering with different steps, including cell entry of the virus life cycle [75]. Arbidol inhibits both LASV and Ebola virus (EBOV) GP-mediated fusion events required to complete the virus cell entry process [76,77]. Screening of a natural product library identified tangeretin as a cell entry inhibitor of seven different HF-causing viruses [78].

Several compounds have been shown to inhibit mammarenavirus cell entry via off-target activities distinct from their intended therapeutic effects. The clotrimazole-derivative TRAM-34 is an ion channel blocker that antagonizes the calcium-activated potassium channel KCa3.1. TRAM-34 specifically inhibited mammarenavirus GP2-mediated fusion, and this anti-mammarenaviral effect was independent of its channel blocking activity [79]. Losmapimod, an p38 MAPK inhibitor, was developed as a therapeutic drug for chronic obstructive pulmonary disease (COPD) and has been reported to inhibit LASV entry by blocking the pH-dependent GP2-mediated fusion without requiring inhibition of p38 MAPK [80]. NH125, a selective eEF-2 kinase inhibitor, inhibited cell entry of recombinant VSVs expressing envelope glycoproteins of avian influenza virus, EBOV, and LASV due to the compound lysosomotropic properties and independently of eEF-2 kinase inhibition [81]. ZCL278 was identified as an inhibitor of Cdc42, a small GTPase that regulates actin polymerization [82]. Subsequently, ZCL278 was shown to exhibit broad-spectrum anti-viral activity, including against mammarenaviruses, by redistributing viral particles from endosomal to lysosomal membranes, and this antiviral activity was not dependent on the downregulation of Cdc42 activity [83]. The antifungal drug isavuconazole, which was approved by the FDA as an orphan drug for aspergillosis, mucormycosis, and candidiasis, was shown to inhibit viral fusion, targeting the SSP-GP2 interface of LASV [84]. The adamantyl diphenyl piperazine 3.3 was developed to target the lysosome-associated membrane protein 1 (LAMP1) by competing with cholesterol, preventing the interaction between LAMP1 and LASV GP [85].

### 3.2. Viral Genome Replication

The nucleoside analog ribavirin has been shown to inhibit mammarenavirus replication in cell culture systems, as well as to have clinical benefits when used to treat LF cases at early times after the onset of clinical symptoms [86,87]. Several different mechanisms of action have been proposed to account for the antiviral activity of ribavirin, including up-regulation of interferon responses [88], GTP pool depletion by inhibiting IMP dehydrogenase (IMPDH), and direct inhibition of viral RNA-directed RNA polymerase (RdRp) activity [89]. Due to its broad-spectrum antiviral activity against multiple RNA and DNA viruses, most likely different mechanisms of action, and combination of them, contribute to the antiviral activity of ribavirin. Notably, a recent study has shown that the main mechanism of the antiviral activity of ribavirin against LASV, is by protecting LASV-infected cells from death [90].

Favipiravir was initially developed as an antiviral drug targeting the influenza virus polymerase and subsequently shown to exhibit a broad-spectrum activity against different RNA viruses, including mammarenaviruses, bunyaviruses, flaviviruses, alphaviruses, picornaviruses, and noroviruses [91,92]. BCX4430 is another broad-spectrum inhibitor of many types of RNA viruses, including mammarenaviruses, which interferes with the activity of the viral RdRp by acting as an RNA chain terminator [93].

Peptide-conjugated phosphorodiamidate morpholino oligomers (PPMO), designed to target conserved regions within mammarenavirus genome RNA, were effective against multiple mammarenaviruses, including JUNV, LCMV, TCRV, and PICV [94]. The inhibitors of DEDDh family of 3′-5′ exonucleases aurintricarboxylic acid (ATA) and pontacyl violet 6R (PV6R) have been reported to inhibit the 3′-5′ exonuclease activity of LASV NP [15], which in addition to its role counteracting the host cell IFN-I response, has been shown to play a critical role in viral fitness in IFN-I deficient cells [95]. As metal-chelating pharmacophores, diketo acids, polyphenols, and *N*-hydroxyisoquinoline-1,3-diones were able to inhibit the endonuclease activity of arenavirus L protein, which resulted in inhibition of the cap-snatching mechanism used by mammarenavirus polymerases to initiate transcription of viral mRNAs [96]. A carboxamide-derivatized disulfide, NSC4492, was reported to show antiviral activity against JUNV and TCRV [97] and this compound was shown to impair viral RNA synthesis of JUNV via targeting the replication complex [98]. KP-146 was shown to have dual roles in its antiviral activity against LCMV, not only interfering with vRNP activity responsible for directing LCMV genome replication and gene transcription but also inhibiting Z protein-mediated budding process [95].

### 3.3. Processing of GPC

The mammarenavirus GPC precursor is co-translationally cleaved by cellular signal peptidases to generate a 58 amino acid-long stable signal peptide (SSP) and the immature GP1/GP2 precursor [99]. Subsequent processing of GP1/GP2 by the cellular subtilisin kexin isozyme-1 (SKI-1)/site-1 protease (S1P) into GP1 and GP2 is required for the production of infectious progeny [17,19]. GP1, GP2, and the SSP form the mature trimeric GP spike complex [99]. Decanoyl-RRLL-chloromethylketone (dec-RRLL-CMK) was developed as a SKI-1/S1P inhibitor based on the cleavage recognition site present within LASV GPC [100] and was shown to exert a potent antiviral activity against LCMV as well as additive antiviral drug activity in combination with ribavirin [101]. PF-429242, a small molecule inhibitor of S1P, was shown to interfere with the proteolytic processing of GPC, which correlated with the compound’s ability of inhibiting multiplication of LCMV and LASV in cultured cells [102]. In S1P-deficient cells, wild-type LCMV consistently underwent extinction without emergence of S1P-independent escape variants [101]. Moreover, PF-429242 efficiently and rapidly cleared persistent virus from infected cells, and interruption of drug treatment did not result in re-emergence of infection, indicating that PF-429242 treatment resulted in virus extinction [103]. These findings indicate a high genetic barrier for the emergence of viral variants capable of using an alternative host cellular protease for the processing of GPC, thus making S1P a very attractive target for the development of antiviral drugs against mammarenaviruses.

### 3.4. Virion Assembly and Cell Egress

Assembly and cell release of infectious mammarenavirus progeny involves Z-L, Z-NP, and Z-GP interactions to facilitate the co-localization of all viral proteins for the assembly of mature infectious particles [30]. Functional studies have shown that the matrix Z protein plays a key role in mammareanvirus budding, a process mediated by the interaction of Z late (L) domain motifs, PTAP and PPPY, with components of the cellular ESCRT complexes [104]. Z-mediated budding also requires myristoylation of the Z protein at a glycine (G) in position 2 to target Z to the plasma membrane, the location of arenavirus budding [105]. Accordingly, treatment with 2-hydroxymyristic acid, an inhibitor of the N-myristoyltransferase (NMT), impaired Z budding activity and production of mammarenavirus infectious progeny [105]. Valproic acid (VPA), a short-chain fatty acid, used in anti-epileptic therapy [106], was shown to inhibit Z-mediated budding of LCMV, likely due to VPA-mediated alteration of the lipid composition of cellular membranes, which is critical in virus budding [107]. Compound 0013, identified as a potent inhibitor of the interaction between the PTAP L domain and Tsg101, a member of the host cell ESCRT complex proteins, was shown to inhibit viral budding by blocking the Z-Tsg101 interaction [108]. The ubiquitin ligase Nedd4 E3 is also a component of the ESCRT complex [109]. A small molecule termed compound1 was identified as an inhibitor of Z-Nedd4 interaction, resulting in inhibition of viral budding [110]. BEZ-235, a phosphatidylinositol 3-kinase (PI3K) inhibitor, was shown to inhibit Z protein-mediated budding of LCMV and LASV by a mechanism of action yet to be determined [111]. In addition to driving virion assembly and release, Z proteins have been reported to modulate various host mechanisms such as repression of translation by binding and counteracting eIF4E [26] and suppression of host innate immune responses [112], suggesting potential host targets for developing antiviral drugs. Recent Z interactome study has identified human proteins that interact with arenavirus Z and validated potential host targets for antiviral therapeutics, including ADP ribosylation factor 1 (ARF1), ATP synthase, H transporting mitochondrial F1 complex beta polypeptide (ATP5B), ATPase H transporting lysosomal 38-kDa V0 subunit d1 (ATP6V0D1), inosine monophosphate dehydrogenase 2 (IMPDH2), peroxiredoxin 3 (PRDX3), and Ras-related protein Rab5c [113].

### 3.5. Monoclonal Antibodies

Monoclonal antibody-based therapies represent an attractive strategy to treat infections by highly pathogenic mammarenaviruses. A monoclonal antibody specific for human transferrin receptor 1 (hTfR1), the receptor used by pathogenic NW mammarenaviruses, inhibits viral entry of several NW mammarenaviruses, including JUNV, GTOV, CHAV, SABV, and MACV [114]. In addition, monoclonal antibodies targeting mammarenavirus GP have been reported to have potent neutralizing activity against MACV and LASV that correlated with inhibition of virus multiplication [115,116]. A recent study documented the isolation of human monoclonal antibodies from LF survivors and characterized their epitope and neutralization profiles, showing that 80% of the monoclonal antibodies with neutralizing activity targeted complex epitopes involving LASV GP1 and GP2 subunits [117]. Importantly, this study identified several human monoclonal antibodies with neutralizing activities against members of the main four lineages of LASV, and some of them showed cross-reactivity to LCMV, LUJO, and MACV. This finding has provided insights to develop therapeutic strategies based on the use of broadly reactive monoclonal antibodies.

### 3.6. Targeting Host Factors

As strict parasites, viruses rely on many host cell factors to complete their life cycles. Therefore, there is increasing interest in targeting host cell factors required for virus multiplication as an antiviral drug strategy. Direct-acting antivirals (DAAs) that target specific viral gene products and functions are likely to be well tolerated by the infected host cell. Still, they are limited by the common problem in antiviral therapy posed by the emergence of drug-resistant variants. In contrast, the emergence of viral variants resistant to host-targeting antivirals (HTAs) is usually significantly reduced or entirely absent, but HTAs can be associated with significant side effects. However, side effects associated with the use of HTAs might be manageable in the case of acute infections, such as HF disease caused by arenaviruses, where the duration of the treatment would be rather short.

Dihydroorotate dehydrogenase (DHODH) small molecule inhibitors A3 and A77172 interfere with de novo pyrimidine biosynthesis and exhibit potent antiviral activity against LCMV and JUNV [118,119]. Likewise, de novo purine biosynthesis is a potential cellular target for the development of HTAs. Inhibition of inosine monophosphate dehydrogenase (IMPDH), a key enzyme in the purine biosynthesis pathway, was shown to be associated with a broad-spectrum antiviral activity against RNA viruses including JUNV [120,121]. S-adenosylhomocysteine hydrolase (SAHH) is an important cellular enzyme for regulating viral mRNA capped structures, and inhibition of SAHH activity by 3-deazaneplanocin was associated with potent antiviral activity against TCRV and PICV [122]. ATPase Na+/K+ transporting subunit alpha 1 (ATP1A1) and prohibitin (PHB) were identified as mammarenavirus NP-interacting host cell proteins and act as pro-viral factors that promote mammarenavirus multiplication. Accordingly, the ATP1A1 inhibitors, ouabain and bufalin, as well as the PHB inhibitor, rocaglamide A, exhibit potent antiviral activity against LCMV and LASV infection [123].

Anti-mammarenaviral drugs discussed in this section have been summarized in Table 1.

## 4. High-Throughput Screening for Discovery of Anti-Mammarenaviral Drugs

The development of mammarenavirus reverse genetics (RG) systems has provided investigators with a novel and powerful approach for the investigation of the cis-acting sequences and trans-acting factors that control arenavirus replication, gene expression, and budding, as well as the rescue of infectious mammarenaviruses from cloned cDNAs. These advances in mammarenavirus molecular genetics have facilitated the generation of recombinant mammarenaviruses expressing reporter genes of interest that have enabled the development of cell-based assays and HTS strategies to identify novel anti-mammarenaviral drugs and assess how they target each of the different steps of the virus life cycle [36]. Different strategies, including the development of tri-segmented mammarenaviruses [36] and the use of the self-cleaving P2A linker to facilitate expression of a reporter gene and NP from the same bi-cistronic NP mRNA [73], have been used to generate these recombinant mammarenaviruses.

However, the use of recombinant infectious HF mammarenaviruses expressing reporter genes for antiviral drug screening campaigns or the investigation of drug mechanism of action would face the complications imposed by the requirement of BSL4 biocontainment. Hence, the advantage of the implementation of RG approaches to develop non-infectious cell-based assays recreating each of the key steps of the virus life cycle and that is amenable to HTS, which can be used without the need of a high-level biocontainment facility. Thus, a number of different platforms have been developed to screen for compounds capable of inhibiting cell entry mediated by GPs of different HF mammarenaviruses, using pseudotype viruses [124,125,126]. Likewise, the biosynthetic processes of replication and transcription of HF mammarenavirus genomes can be mimicked using cell-based minireplicon, or minigenome (MG) systems [127]. These cell-based MG systems are based on the intracellular reconstitution of a functional vRNP directing expression of a reporter gene whose expression level serves as a surrogate of the vRNP activity. For this, cells are transfected with plasmids expressing the viral trans-acting factors L and NP, together with a plasmid that allows for intracellular synthesis of an RNA containing the open reading frame of a reporter gene under the control of the cis-acting regulatory sequences of the S or L genome RNA [54]. In addition to these transient transfection-based MG systems, cell lines have been generated to constitutively express a functional, non-infectious vRNP of LCMV or LASV [128]. This overcomes some technical complications related to transient transfection in the context of HTS. Thus, cell lines expressing LCMV and LASV functional vRNPs were successfully used to screen different compound libraries, resulting in the identification of a number of hits that were confirmed to exhibit antiviral activity against infectious LCMV and LASV [128].

Generation of infectious virus-like particle (iVLP) systems containing functional virus MGs allows for modeling of not only viral genome replication but also cell entry and budding [129]. A chimeric protein consisting of a secretion deficient form of *Gaussia* luciferase (GLuc) fused to the C-terminus of LASV Z protein was successfully used to develop a cell-based assay to quantify Z-mediated budding activity. This assay has features compatible with its use in HTS [130], as levels of GLuc in the tissue culture supernatant serve as an accurate surrogate of Z budding activity.

## 5. Drug Repurposing Strategy

The Discovery and development of novel drugs require significant investments and resources and an average processing time for market authorization of 10 to 17 years [131]. The rapid development of antiviral therapeutics is important to combat emerging viruses. Finding novel applications of clinically approved drugs can accelerate the drug development process and significantly reduce risks during clinical trials assessing the new drug application. Accordingly, repurposing existing drugs is considered an attractive strategy to combat emerging viral infections [132]. This has been illustrated by efforts to combat the current COVID-19 pandemic, where screening of libraries of already approved drugs resulted in the rapid identification of anti-SARS-CoV-2 drug candidates that were very rapidly advanced to clinical trials [133]. Among the listed compounds in Table 1, ribavirin, arbidol and favipiravir are currently being tested in COVID-19 patients in clinical trials [133].

Screening of a library of FDA-approved drugs using VSV pseudotyped with LASV GP identified a number of inhibitors of LASV GP-mediated cell entry [125,134]. Likewise, screening of the Repurposing, Focused Rescue, and Accelerated Medchem (ReFRAME) library identified several potent anti-mammarenaviral compounds [135]. Importantly, selected hits initially identified based on their anti-LCMV activity, which were confirmed to show potent antiviral activity against the HF causing mammarenaviruses LASV and JUNV. These compounds exerted their antiviral activity via targeting host cellular factors, including enzymes required for pyrimidine and purine biosynthesis, regulators of apoptosis, and the mitochondrial electron transport complex III [135]. Recently, this ReFRAME library was used to screen for antiviral drugs against SARS-CoV-2, and the existing pharmacological and safety data on the identified hits will facilitate their rapid testing in the clinic [136]. In addition to HTS formats to rapidly identify novel targets and antiviral drug candidates, function-focus based assays have also been successfully used to identify compounds that could be repurposed as antiviral drugs. For example, screening of a collection of kinase inhibitors identified several cellular kinases that were involved in LASV GP-mediated viral entry, including protein kinase C, phosphoinositide 3-kinase, and human hepatocyte growth factor receptor (HGFR), which is a receptor tyrosine kinase [137].

## 6. Conclusions and Future Perspectives

As documented in this review, significant efforts have been dedicated to finding effective antiviral drugs against human pathogenic mammarenaviruses. Different screening platforms have identified a number of antiviral drug candidates with potent activity in cell-based infection assays. However, for the majority of the identified hits, there is only very limited information regarding their in vivo efficacy. To advance the development of novel effective antiviral drugs, further validation should be conducted using appropriate in vivo models of mammarenavirus disease, including non-human primates.

Drug repurposing approaches have identified a number of host cell factors as attractive antiviral targets for which drugs with a good safety profile have been already documented, which should facilitate the assessment of their efficacy in vivo using appropriate animal models of mammarenavirus induced disease. Since different viruses may share some key host cell functions to complete their life cycle, a host-targeting strategy would be an attractive approach for the development of broad-spectrum antiviral therapeutics. Synergistic antiviral effects have been documented in combination therapies of approved drugs, as illustrated by the results of combination therapy of ribavirin and favipiravir against LASV infection in pre-clinical [87] and clinical [138] studies. Likewise, combination therapy of arbidol with aripiprazole or sertraline resulted in synergistic inhibition of pseudotyped viruses with GPs from LASV and JUNV [139]. Synergistic effects are likely to be facilitated by combination therapy with drugs targeting different steps of the virus life cycle. To identify combinations for antiviral therapeutics, modern computational approaches, including available data libraries and analytical resources [140], would be promising tools by which data and text mining could identify potential drug combinations for further experimental validations.

## Figures and Tables

**Table 1:** Drugs with anti-mammarenaviral activity.

Target	Drug Name	Mechanism
Viral entry	ST-193	- GP2 targeting compounds - Inhibition of pH-dependent membrane fusion
	ST-294	
	ST-336	
	LHF-535	
	F3406	
	AVP-p	
	arbidol	
	tangeretin	
	TRAM-34	- Calcium-activated potassium channel blocker - Inhibition of pH-dependent membrane fusion
	losmapimod	- p38 MAPK inhibitor - Inhibition of pH-dependent membrane fusion in p38 MAPK down regulation-independent manner
	NH125	- eEF-2 kinase inhibitor - GP-mediated fusion inhibition due to lysosomotropic properies
	ZCL278	- Cdc42 inhibitor - Fusion inhibition by redistributing viral particles from endosomal to lysosomal membranes
	isavuconazole	- Antifungal drug for aspergillosis - Inhibition of pH-dependent membrane fusion by targeting the SSP-GP2 interface
	dec-RRLL-CMK	- Blocking the proteolytic processing of GPC by inhibition of host protease S1P
	PF-429242	
	adamantyl diphenyl piperazine 3.3	- Blocking the interaction between host LAMP1 and viral GP
Viral genomereplication	ribavirin	- Upregulation of host IFN responses - Cellular GTP depletion - Viral RdRp inhibition
	favipiravir	- Viral RdRp inhibition
	BCX4430	
	PPMO	- Interfering with viral RNA synthesis and translation
	ATA	- Inhibition of NP exonuclease activity
	PV6R	
	diketo acids	- Inhibition of L endonuclease activity
	polyphenols	
	N-hydroxyisoquinoline-1,3-diones	
	NSC4492	- Targeting the vRNP to impair viral RNA synthesis
	KP-146	
	A3	- DHODH inhibitor - Inhibition of de novo pyrimidine biosynthesis
	A771726	
	acridone	- IMPDH inhibitor - Inhibition of de novo purine biosynthesis
	bredinin	
	3-deazaneplanocin	- Inhibition of SAHH activity which is important in viral mRNA capped structure
	ouabain	- ATP1A1 inhibitor - Preventing interaction with viral NP
	bufalin	
	rocaglamide	- PHB inhibitor - Preventing interaction with viral NP
Virion assembly and budding	2-hydroxymyristic acid	- Inhibition of NMT which mediates myristoylation of Z protein - Blocking Z-mediated budding
	valproic acid	- Altering lipid composition of cellular membranes which is critical in virus budding
	compound0013	- Tsg101 inhibitor - Blocking interaction between Tsg101 and viral Z protein that is required for virion egress
	compound1	- Inhibiting the interaction between Nedd4 and Z protein, blocking viral budding
	BEZ-235	- PI3K inhibitor - Inhibition of Z-mediated budding via unknown mechanism
	KP-146	- Dual roles in viral genome replication and budding
